# Comprehensive Profiling of lincRNAs in Lung Adenocarcinoma of Never Smokers Reveals Their Roles in Cancer Development and Prognosis

**DOI:** 10.3390/genes8110321

**Published:** 2017-11-13

**Authors:** Ying Li, Zheng Wang, Asha Nair, Wei Song, Ping Yang, Xiaoju Zhang, Zhifu Sun

**Affiliations:** 1 Department of Health Sciences Research, Mayo Clinic, Rochester, MN 55905, USA; 13523436663@126.com (Y.L.); nair.asha@mayo.edu (A.N.); yang.ping@mayo.edu (P.Y.); 2 Department of Pulmonary Medicine, People’s Hospital of Henan Province, Zhengzhou 450003, China; santawang99@163.com (Z.W.); sw65229581@126.com (W.S.)

**Keywords:** long intergenic non-coding RNA, never smokers, lung adenocarcinoma, RNA-sequencing

## Abstract

Long intergenic non-coding RNA (lincRNA) is a family of gene transcripts, the functions of which are largely unknown. Although cigarette smoking is the main cause for lung cancer, lung cancer in non-smokers is a separate entity and its underlying cause is little known. Growing evidence suggests lincRNAs play a significant role in cancer development and progression; however, such data is lacking for lung cancer in non-smokers, or those who have never smoked. This study conducted comprehensive profiling of lincRNAs from RNA sequencing (RNA-seq) data of non-smoker patients with lung adenocarcinoma. Both known and novel lincRNAs distinctly segregated tumors from normal tissues. Approximately one third of lincRNAs were differentially expressed between tumors and normal samples and most of them were coordinated with their putative protein gene targets. More importantly, lincRNAs defined two clusters of tumors that were associated with tumor aggressiveness and patient survival. We identified a subset of lincRNAs that were differentially expressed and also associated with patient survival. Very high concordance (*R^2^* = 0.9) was observed for the differentially expressed lincRNAs in the Cancer Genome Atlas (TCGA) validation set of 85 transcriptomes and the lincRNAs associated with survival from the discovery set were similarly predictive in the validation set. These lincRNAs warrant further investigation as potential diagnostic and prognostic markers.

## 1. Introduction

Lung cancer is the leading cause of cancer death worldwide. Although lung cancer mostly occurs in patients with a smoking history, the incidence in those who have never smoked is increasing [1,2,3], which approximately accounts for 25% of lung cancer patients and would rank as the seventh most common cause of cancer mortality around the world [4]. Lung cancer in non-smokers has many unique clinical, pathological and genetic features, which include dominant adenocarcinoma subtype, more frequent in women, higher frequency of *EGFR* and *EML4-ALK* mutations, and family history of lung cancer [2,3]. These characteristics make it a different disease and studies specific to this entity are needed [4].

Long noncoding RNAs (lncRNAs) are a large class of gene transcripts discovered or characterized in recent years [5,6] and those located in intergenic regions (lincRNAs) are a subset that can be detected from diverse RNA sequencing (RNA-seq) protocols as they have no overlap with other transcripts and overlapping transcripts need to be distinguished by strand specific RNA-seq. Accumulating evidence suggests that lncRNAs or lincRNAs play a significant role in cancer etiology and progression and may be potential diagnostic and prognostic markers for various cancers [5]. Some of well-known lncRNAs related to lung cancer etiology and progression are well described [7,8,9]. MALAT1, CCAT1, H19, and UCA1 are reported to be oncogenic and associated with worse outcome; MEG3, TUG1 BANCR, and GAS5 are tumor-suppressive and associated with better outcome in lung adenocarcinoma [7].

With the popularity of RNA-seq, genome-wide view of lincRNAs is emerging. Through mining 567 adenocarcinoma and squamous cell carcinoma tumors with normal lung controls, a study reported 111 “lung cancer-associated lncRNAs” [9]. However, this patient cohort is mostly from smokers and no information is available for those who have never smoked.

Taking advantage of a well-characterized lung cancer cohort from ‘never smokers’ with RNA-seq data at our institution, we conducted comprehensive lincRNA profiling for 27 pairs of tumor and normal transcriptomes. Not only did we profile known lincRNAs, we also detected and analyzed predicted novel lincRNAs using our internally developed pipeline. For both known and novel lincRNAs we identified tumor associated lincRNAs and lincRNAs associated with patient survival. These findings were further validated in an independent cohort of never-smoker patients with lung cancer from the Cancer Genome Atlas (TCGA).

## 2. Materials and Methods

### 2.1. Never-Smoker Patients with Lung Adenocarcinoma

The cohort consisting of 27 patients with lung adenocarcinoma from never smokers was described in our previous publication [10] and the paired tumor and normal tissues were sequenced by RNA-seq. Briefly, all participants were provided written informed consent, and the study protocols were approved by the Mayo Clinic Institutional Review Boards (IRB#225-99). A pair of fresh frozen tissue, one from the primary tumor and another from the unaffected normal lung, was cut, stained, and reviewed by an experienced pathologist to make sure each case’s correct diagnosis and sufficient tumor component (greater than 70%) to be qualified for the study. Included patients in this study all had adenocarcinoma and were in stage I. The follow-up time ranged from 2.98 to 11.94 years with the median time of six years.

### 2.2. RNA-Sequencing Data and Long Intergenic Non-Coding RNA Detection

The raw sequencing data was deposited into Gene Expression Omnibus (GEO accession GSE87340). Briefly, total RNA extraction was performed using Exiqon’s miRCURY RNA Isolation Kit (Exiqon, Vedbaek, Denmark). mRNA was resolved using poly-dT oligonucleotides attached to magnetic beads (Illumina, San Diego, CA, USA), fragmented using divalent cations under elevated temperatures, and converted to complementary DNA (cDNA) using random primers. After conversion of the cleaved fragments into cDNA, the cDNA underwent blunt end repair, addition of an ‘A’ base to the 3′ blunt ends, and ligation of adapter molecules which were used for PCR amplification, and bridge amplification. The sequencing was carried out using the Illumina HiSeq 2000 sequencer (Illumina, San Diego, CA, USA) (pair-end in 50 bp long). For lincRNA detection, both known and novel, we used our internally developed pipeline Ultrafast and Comprehensive long non-coding RNA detection from RNA-seq (UClncR) [11]. Briefly, the Tophat [12] aligned bam files (version 1.4.0) were provided to UClncR, which conducts transcript assembly using StringTie [13] and novel lincRNA prediction after various filtering steps (coding length >200 bases, protein coding potential <0.1, expression level >10 percentile, and overlap with repetitive region less than 5%). The predicted novel lincRNAs from each sample were merged across all samples using Cuffmerge function in Cufflinks [14]. This master transcript annotation was then combined with known lincRNAs defined in GENECODE (v18) annotation for gene expression quantification by featureCounts (v.1.4.6) [15]. 

### 2.3. Long Intergenic Non-Coding RNA Differential Expression between Tumors and Normal Lungs

Differential gene expression was conducted between tumors and their normal lung tissues on raw gene count using DESeq2 [16], in which the normalization was performed by RLE algorithm (Relative Log Expression) and differential expression by negative binomial general liner model and Wald statistics on the two groups (unpaired). LincRNAs with false discovery rate (FDR) <0.05, mean expression value >1 and absolute log_2_ fold change >1 were defined as significant. For analyses that compared relative expression across genes or unsupervised analysis such as principal components analysis (PCA) or unsupervised clustering, log_2_ RPKM (reads per kilobase per million mapped reads) normalized data [17] was used, which was conducted on all lincRNAs together with the total number of reads mapped to lincRNAs in a sample (as library size), lincRNA coding length, and expression value of each lincRNA in a sample.

As the biological functions for vast majority of lincRNAs are unknown, we interpreted differentially expressed or patient survival associated lincRNAs using their neighboring protein coding genes if they were within 10 kb upstream and 1.5 kb downstream of the transcription start site (TSS) of the protein coding gene. Pathway enrichment analysis was performed by Ingenuity Pathway Analysis (IPA) (https://www.qiagenbioinformatics.com/products/ingenuity-pathway-analysis/).

### 2.4. Long Intergenic Non-Coding RNA Association with Clinical Variables and Patient Survival

For the tumor samples, we conducted unsupervised clustering using all lincRNAs (either known or novel) and the clusters formed were evaluated for their association with clinical variable of patient age at diagnosis (grouped into young, intermediate and old groups), gender, tumor stage, and histologic grade of differentiation by Fisher’s exact test (*t* test was also conducted for age at diagnosis between two clusters). The significant variables from univariate analysis were also included for multivariate analysis using Cox proportional hazards model. Kaplan Meier survival curves and log rank test were used for survival difference between groups.

### 2.5. Validation Dataset from the Cancer Genome Atlas

From TCGA lung adenocarcinoma dataset, we identified those who did not have smoking history. We downloaded the raw RNA-seq data and aligned them to human reference genome (hg19) by HiSAT2 [18], an improved much faster version of Tophat. The known and novel lincRNAs were quantified by featureCounts [15] after combining the known lincRNAs with the predicted novel lincRNAs from the internal discovery dataset of 27 tumor/normal pairs. These lincRNAs were analyzed in the similar manner as the internal dataset.

All analyses were performed using R (v3.2.3, https://www.r-project.org/) and relevant Bioconductor packages (http://bioconductor.org/)

## 3. Results

### 3.1. Known Long Intergenic Non-Coding RNA Expression between Tumor and Normal Lung Samples

**Figure 1:**
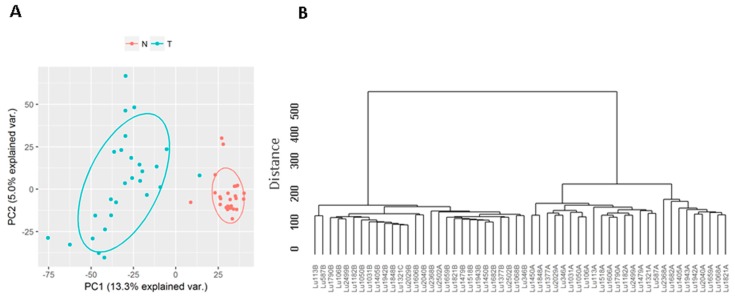
Principal components analysis (PCA) and unsupervised clustering by known long intergenic non-coding RNAs (lincRNAs). (**A**) PCA shows that all tumors except 1 form a distinct cluster from the normal tissues; normal lung tissues (N); lung tumors (T); (**B**) Unsupervised clustering shows the similar pattern. Both suggest the tumors and normal tissues have distinct lincRNA transcription programs.

Among the 6763 known lincRNAs, 6248 had at least 1 read expressed in one of the 54 tumor and normal samples. PCA and unsupervised clustering analysis showed the tumors and normal tissues formed two distinct clusters, suggesting they had very different lincRNA expression profiles (Figure 1).

**Figure 2:**
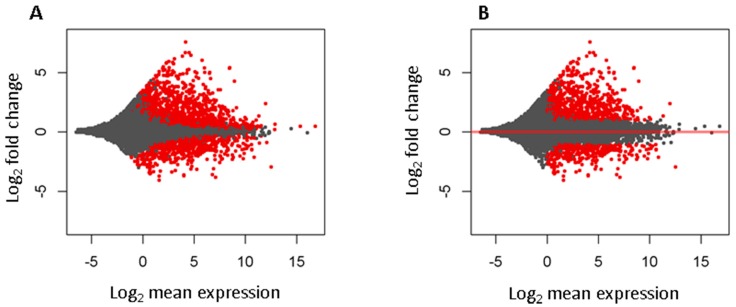
M–A (log ratio vs. mean) plot for differentially expressed known lincRNAs between tumors and normal lung tissues. *X*-axis for mean expression of a lincRNA across all samples and *y*-axis for log_2_ fold changes comparing tumor to normal samples. Each dot represents a gene. Genes with red color are significantly changed. (**A**) Changed lincRNAs at false discovery rate (FDR) <0.05; (**B**) Changed lincRNAs with FDR <0.05, log_2_ fold change greater than 1 (2 fold), and mean expression of reads per kilobase per million mapped reads (RPKM) value greater than 1.

Differential expression analysis found 1708 significantly changed lincRNAs at false discovery rate less than 0.05, among which 1111 were up and 597 down expressed in tumors. To select more reliable and biologically meaningful lincRNAs, we further filtered out those low expressed (mean expression <1) and with absolute log_2_ fold change less than 1, which led to 1067 (701 up and 366 down expressed in tumors) lincRNAs (Figure 2, and Supplementary Table S1).

### 3.2. Long Intergenic Non-Coding RNA and Potential Target Protein Coding Gene Association

**Figure 3:**
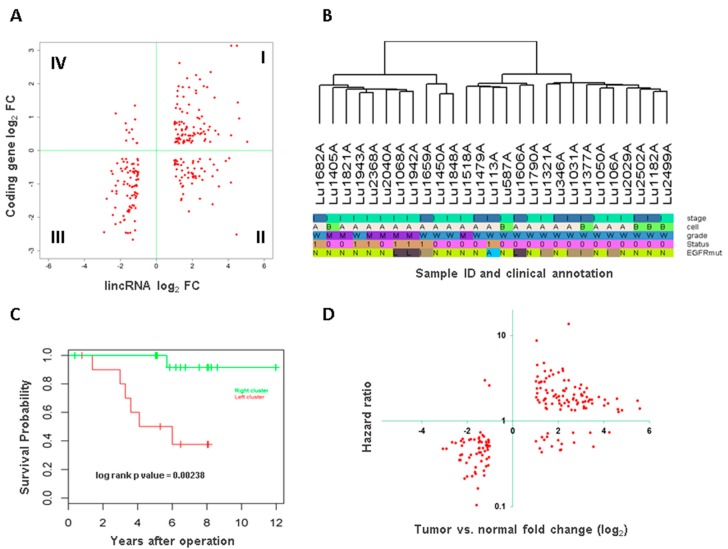
LincRNA association with protein coding genes and clinical variables. (**A**) Fold change (FC) comparison of differentially expressed lincRNAs and their adjacent protein coding genes. Most of them are in coordinated changes, i.e., both either up or down expressed as the dots shown in quadrant I and III; (**B**) Unsupervised clustering with clinical variables. Stage: dark blue for stage IA and light green for stage IB; Cell: A—adenocarcinoma, B—adenocarcinoma with bronchoalveolar carcinoma feature (or lepidic predominant adenocarcinoma in the new classification); Grade: W—well differentiated and M—intermediately differentiated; Status: patient survival status, 1—dead and 0—alive; EGFRmut: *EGFR* mutation status, N—none, L—L858R substitution, I—insertion/deletion, A750P substitution; (**C**) Kaplan-Meier survival curves by clusters defined by lincRNA expression; (**D**) lincRNA differentiation and association with patient survival. lincRNAs that were highly expressed in tumors also led to worse survival (positive hazard ratio). Conversely those expressed lower had reduced risk to patient survival.

As most lincRNAs have no known functions, one of the common approaches is to look their association with nearby protein coding genes for their potential regulation or association. For this we checked the differential expression data of protein coding genes and those differentially expressed lincRNAs within 10 kb upstream or 1.5 kb downstream of protein coding gene transcription start sites. This analysis identified 250 lincRNA and protein coding gene pairs that were both differentially expressed. Interestingly, the vast majority of these pairs (180, 72%, Χ^2^ *p* value = 1.602 × 10^−14^) demonstrated coordinated expression, i.e., the change of lincRNA was in the same direction as its neighboring protein coding gene (Figure 3A). Pathway analysis for these protein coding genes showed significantly enriched pathways such as ´signaling by rho family GTPases´, ´notch signaling´, ´protein kinase A signaling´, and ´integrin signaling´ (Supplementary Table S2).

### 3.3. Association of Long Intergenic Non-Coding RNA with Tumor Aggressiveness and Patient Survival

Unsupervised clustering was performed for the tumor samples by all known lincRNAs and two distinct clusters were observed (Figure 3B). The clusters were significantly associated with tumor histological grade of differentiation (Fisher’s exact test *p* value = 0.009) but none of other variables (age at diagnosis, gender, tumor stage, cell subtype and *EGFR* mutation status). Kaplan Meier survival analysis showed the tumors in the left cluster had significantly worse survival than those in the right cluster (log rank test *p* value = 0.002, Figure 3C). Multivariate analysis with age, gender, and tumor grade as covariates in the model showed the cluster separation remained as an independent predictor for survival (*p* value = 0.02).

### 3.4. Long Intergenic Non-Coding RNAs Associated with Both Cancer Development and Patient Survival

As tumor clusters by lincRNAs were associated with patient survival, we were interested in which were these lincRNAs. Furthermore, lincRNAs that were both differentially expressed between tumor and normal samples and also significantly associated with patient survival were likely key players in carcinogenesis and tumor progression. To this end, we conducted survival association analysis for all known lincRNAs by Cox proportional hazards model and those with significant association with overall survival (*p* value < 0.05) were compared with the differentially expressed lincRNAs identified by the previous step. This analysis identified 169 lincRNAs (Supplementary Table S3). Intriguingly, majority of these lincRNAs showed positive relationship between differential expression and hazard ratio of the survival, i.e., lincRNAs that were highly expressed in tumors also increased the risk of patient death and those that were lower in tumors had a protective effect (Figure 3D). The lincRNAs in the first category are more likely oncogenes while the latter may play a tumor suppressor role. For example, *RAMP2-AS1*, which had the lowest hazard ratio of 0.1 and was down expressed 3 fold in tumors, was previously reported having tumor suppressor role in glioblastoma [19].

### 3.5. Novel Long Intergenic Non-Coding RNAs and Their Clinical Associations

**Figure 4:**
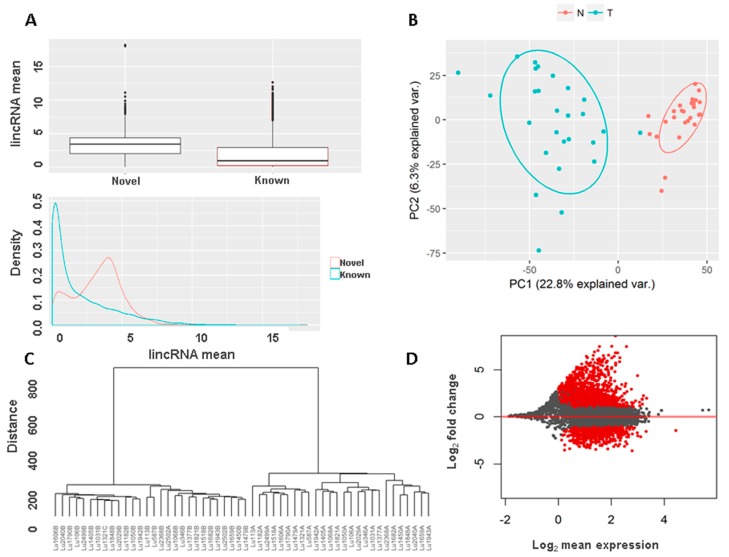
Novel lincRNA profiles. (**A**) Comparison of novel lincRNA expression with known lincRNAs. Overall novel lincRNAs have higher expression than known lincRNAs; (**B**) Novel lincRNAs segregate tumors from normal samples, N for normal and T for tumor samples. (**C**) Unsupervised clustering by novel lincRNAs separated tumors from normal; (**D**) Differentially expressed novel lincRNAs between tumors and normal lungs (highlighted in red).

We detected 5086 novel lincRNAs from this set of 54 transcriptomes, 3704 with single exon and 1382 with multi-exons. The mean expression of these novel lincRNAs across all samples was slightly higher than known lincRNAs (Figure 4A, the same lowest mean was used to filter out low expressed known lincRNAs as the novel lincRNAs), which is not surprising as to name a novel lincRNA certain expression threshold needs to be met for each lincRNA while known lincRNAs with low expression were simply quantified. Principal components analysis by novel lincRNAs also showed clear separation between tumors and normal (Figure 4B). Unsupervised clustering also separated tumors from normal samples (Figure 4C and Figure S1). Differential expression analysis using the same criteria as known lincRNAs found 1802 lincRNA that were significantly deregulated, 1203 up and 599 down in tumor samples, the similar pattern as the known lincRNAs (Figure 4D, Supplementary Table S4). Among these changed novel lincRNAs, 342 were located within 10 K upstream and 1.5 Kb downstream of TSS of 331 protein coding genes. Pathway analysis for these genes showed significant enrichment of canonical pathways like ´G-protein coupled receptor signaling´, ´Wnt/Ca^2+^ signaling pathway´, ´protein kinase A signaling´, and ´Gs α subunit (G_αS)_ signaling´ (Supplementary Table S5).

**Figure 5:**
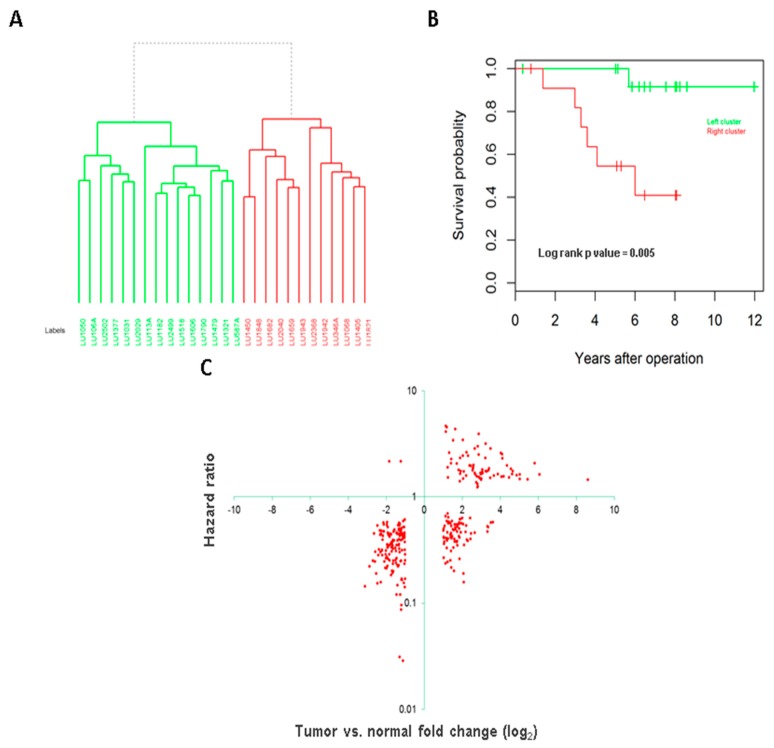
Novel lincRNA expression and association with clinical phenotypes. (**A**) Unsupervised clustering separates tumors into two distinct clusters; (**B**) The two clusters have different survivals by Kaplan Meier analysis; (**C**) Differential expression fold change and hazard ratio of survival association.

Unsupervised clustering by the novel lincRNAs also separated tumors into two clusters (Figure 5A). Comparing to the clusters by known lincRNAs, only one sample (LU346A) switched from one to another cluster. Consistent with the known lincRNAs, the segregation was significantly associated with patient survival (log rank *p* value = 0.005, Figure 5B). The significant remained after adjusting for age, sex and tumor grade (*p* value = 0.02).

Similarly, we conducted survival association analysis for all the novel lincRNAs and found 926 (15.2%) were significantly associated with survival (at *p* value < 0.05). Comparing these significantly associated lincRNAs with the differentially expressed lincRNAs found 301 in common (Supplementary Table S6). As observed in known lincRNAs, most of these lincRNAs were in the positive correlation between differential expression and survival risk. For the lincRNAs that were highly expressed in tumors, the higher expression also gave rise to worse survival for the patients. Conversely, for these lincRNAs that were down-expressed in tumors, the higher expression in the tumors would lengthen patient survival time (Figure 5C).

### 3.6. Validation of Long Intergenic Non-Coding RNAs in the Cancer Genome Atlas Data

**Figure 6:**
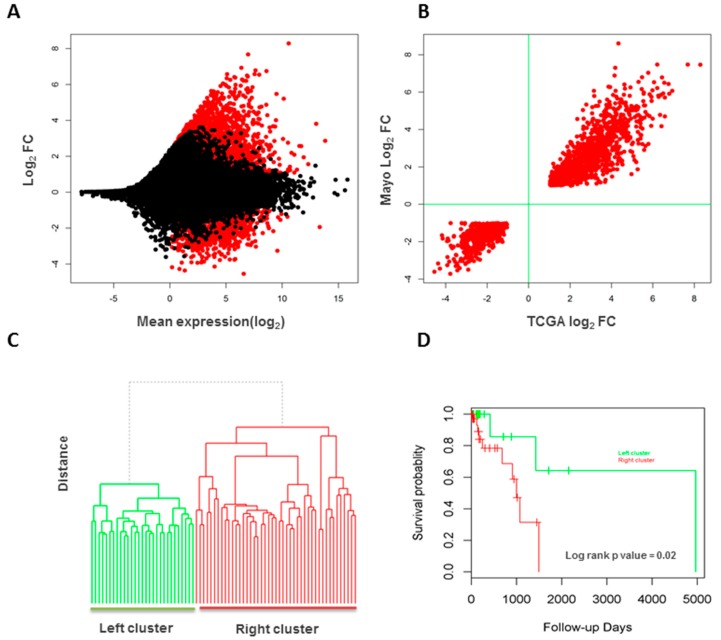
LincRNA validation in the Cancer Genome Atlas lung adenocarcinoma of never-smokers. (**A**) Differential expression between tumors and normal samples. Those significantly changed lincRNAs (red) are more up-expressed; (**B**) The commonly changed lincRNAs between the internal and TCGA datasets are in good agreement for deregulation directions and fold changes; (**C**) Two clusters of tumor samples by lincRNAs; (**D**) The two clusters are associated with patient overall survivals.

For the same set of lincRNAs (both known and novel) analyzed for the 27 tumor/normal pairs, we found 11,415 were detectable in the 85 TCGA lung adenocarcinoma and normal samples (78 tumors and 7 normal lung tissues). PCA and unsupervised clustering segregated tumors from normal samples clearly. Differential expression analysis identified 2049 lincRNAs that were differentially expressed (Figure 6A), of which 1450 (519 known and 931 novel lincRNAs) overlap with the significantly changed lincRNAs from the internal discovery dataset (70.8%, Χ^2^ *p* value < 2.2 × 10^−16^). These commonly changed lincRNAs were in high agreement in terms of change directions (100% consistency) and estimated fold changes (correlation coefficient *R^2^* = 0.9, Figure 6B). Of note again, vast majority of these lincRNAs (71.2%) were highly expressed in the tumors. 

Unsupervised clustering showed two distinct clusters for tumors samples (Figure 6C), which was also significantly associated with patient survival (log rank test *p* value = 0.02, Figure 6D). Using 470 lincRNAs that were differentially expressed and also associated with patient survival in the internal dataset of 27 patients, the 78 tumors were clustered into three groups, which was significantly associated with patient survival status (Fisher’s exact test *p* value = 0.02). Among the lincRNAs that were differentially expressed and associated with patient survival in the internal dataset, we also found 27 (10 known and 17 novel lincRNAs, Supplementary Table S7) that were differentially expressed and significantly associated with patient survival in this TCGA dataset. Notably among the known ones, UCA1 has been reported to promote the growth and metastasis of lung cancer [20], tumor proliferation and 5-fluorouracil resistance of colorectal cancer [21], and tumor growth of breast cancer [22] and bladder cancer [23]. This lincRNA was highly expressed in both our internal and TCGA datasets and was significantly associated with poor survival of patients.

## 4. Discussion

Lung cancer is the number one killer among all cancers. While cancer types related to smoking are declining, lung cancer from non-smokers is increasing and are becoming a burden to society. The distinct clinical, pathological and molecular features of such cancer suggest it may have a different etiology and specific research about this entity might reveal more insights to its development, progression and clinical outcomes. 

As a large family of transcripts discovered in recent years, lncRNAs are attracting growing attention as they may play a significant role in disease development and hold higher hope for novel biomarker discovery [5,7,8]. LincRNAs are non-coding transcripts coded from intergenic regions between two protein coding genes in the genome. As they do not overlap with other transcripts, it is relatively easier to quantify known lincRNAs or detect novel ones from RNA-seq data than lncRNAs that overlap with other transcripts. Although many lincRNAs have been recently annotated, their exact biological functions and underlying molecular mechanisms for vast majority remain unknown. 

This study analyzed a cohort of lung adenocarcinoma patients from never smokers whose clinical data had been well annotated. We conducted comprehensive lincRNA discovery and analysis and revealed many interesting findings. First, as previously noted, lincRNA expression is quite tissue or disease specific. Over 5000 novel lincRNAs were detected from this dataset. Second, both known and novel lincRNAs were highly differentially expressed between tumors and normal samples where tumors appeared having increased expression for majority of lincRNAs. This suggests that tumors may have new lincRNAs expressed or existing lincRNAs in normal lung tissues have increased expression. New cells such as immune cells or fibrotic cells may also contribute to the difference. By comparing differentially expressed lincRNAs and their adjacent protein coding genes, we also found they changed in a coordinated fashion. More importantly, lincRNA expression appeared to be associated with tumor aggressiveness and patient outcome of overall survival. Notably, we found a set of lincRNAs that were both differentially expressed and associated with patient survival in both discovery and validation datasets. Among these, 10 were known and 17 were novel lincRNAs. UCA1 is an interesting one as it was highly expressed in both our and TCGA datasets and was significantly associated with poor survival of patients. It has been previously reported that increased expression of this lincRNA promotes the growth and metastasis of lung cancer [19] and other cancers [20,21,22]. No reports have been found for other lincRNAs and further studies to these lincRNAs are warranted.

LncRNAs have several modes of action as learned from a few well-studied lincRNAs [24]. The most common one is to target their nearby protein coding genes and regulate their expression. HOTAIR and MALAT1 probably are the two most comprehensively studied cancer related lncRNA, whose increased expression are associated with lung cancer progression, metastasis, chemo-resistance and relapse, resulting in patients’ poor prognosis and survival [25,26]. HOTAIR is an antisense lncRNA that would need strand specific RNA-seq for accurate detection while MALAT1 is lincRNA. MALAT1 is highly expressed in tumors of our lung cancer dataset with 1.4× fold change; however, its expression is not associated with patient survival. In TCGA dataset, this gene is not differentially expressed between tumor and normal (*p* value = 0.07) although it is in the same trend of higher expression in tumors. It is not associated with patient survival either. As reported, CCAT1 was up-regulated in both our and TCGA datasets although it was significantly associated with survival only in TCGA dataset, which was likely caused by the underpowered sample size in our dataset. While MEG3 was not differentially expressed in our dataset, it was upregulated in TCGA dataset. BANCR was down-expressed in both our and TCGA dataset although negative survival association was only seen in our dataset. Unlike previously reported, TUG1 was not differentially expressed in our and TCGA dataset. As most of lung cancer are smoking related, studies have been conducted on the transcriptome of lung cancer patients with smoking history revealing broad differences in lncRNA expression pattern between lung cancer tissues and healthy counterparts [9,27,28]. However, data for genome-wide lincRNA profiling in never-smoker lung cancer is very limited. The only available study so far analyzed 3 pairs of tumor and normal tissues and reported 182 differentially expressed lncRNAs, of which 109 were up and 73 were down expressed in tumors [29]. Unfortunately, the paper did not provide the identifiers of these lincRNAs or their genomic coordinates, which makes it impossible to compare. The small sample size is also difficult to get reliable results and capture the heterogeneity of the cancer from different individuals. In this study we did not conduct ´novel prediction´ for known lincRNAs, but rather, quantified them according to known annotation for several reasons. Known lincRNAs represent years of previous work with more evidence support. They have universal identifiers for others to cross link data from different sources. Pure digital prediction for these lincRNAs from RNA-seq data can be limited by sequence depth, data quality or algorithms, which lead to incorrectness or broken structure. Because of these, we analyzed known and novel lincRNAs separately so that we can use known lincRNAs as reference to evaluate the reliability of the predicted novel lincRNAs. Indeed, our data showed that the novel lincRNAs recapitulated the expression profiles and clinical associations of known lincRNAs.

The commonly used RNA-seq protocols are PolyA selection for mRNAs and ribosomal RNA (rRNA) depletion for all remaining RNAs. The former has been more commonly used as it is simpler and more consistent but the latter is getting more popular as it can capture additional transcripts (genes) that are not PolyA tailed [30]. The issue with the latter is that it may contain many intronic reads from un-processed transcripts (pre-messenger RNAs) [31] or contaminated by unwanted rRNAs as a result of insufficient rRNA removal. Both our data and TCGA data were generated from PolyA selection and un-stranded protocol. In lncRNA detection, PolyA protocol obtained the similar number of lncRNAs as rRNA depletion methods and 15–20% lncRNAs were unique to each protocol [31]. LincRNAs can be detected by both protocols equally well [31]. Whether overlapping lncRNAs can be detected depends on stranded or un-stranded protocol, but not RNA enrichment method of PolyA selection or rRNA depletion. As non-stranded RNA-seq protocol cannot separate overlapping transcripts, the current practice is to look lincRNAs only [9,32].

One of the limitations of this study is the relative small sample size of the discovery cohort for survival association, although differentially expressed lincRNAs relative to normal lung tissues are very robust. Survival association is often complicated by heterogeneous patient population. This study limited to stage I disease, which minimized the stage impact, the single most important predictor for patient survival. Although TCGA validation dataset has relatively a larger sample size, the cohort is more diverse as they are from various stages and different institutions. Moreover, many patients have very short follow-up times and observed events of death are low (13 events and 19 patients with follow-up time less than 30 days). Further studies by a large sample size are needed to validate the prognostic lincRNAs as potential biomarkers.

Strictly speaking, the novel lincRNAs predicted from this study should be called novel lincRNA candidates as further validation is needed to assess if they are true and reliable transcripts, particularly for single exon lincRNAs. LincRNAs in adenocarcinoma of never smokers are the focus of this study; however, the next interesting question is whether and how they differ from smoking related adenocarcinoma, which is the direction in the future work.

In summary, our study has identified many lincRNAs, both known and novelly predicted, from lung adenocarcinoma of never smoker individuals that may play a significant role in cancer development, progression, and patient prognosis. 

## Supplementary Materials

The following are available online at www.mdpi.com/2073-4425/8/11/321/s1. Figure S1: Heatmap by differentially expressed novel lincRNAs separating tumors (red bar) from normal samples (green bar); Table S1: Differentially expressed known lincRNAs between 27 pairs of tumors and normal tissues; Table S2: Enriched pathways for protein coding genes that were differentially expressed where their neighboring known lincRNAs were also differentially expressed; Table S3: Known lincRNAs that are differentially expressed and also associated with patient survival; Table S4: Differentially expressed novel lincRNAs between tumors and normal samples; Table S5: Enriched pathways for protein coding genes that were differentially expressed where their neighboring novel lincRNAs were also differentially expressed; Table S6: Novel lincRNAs that are differentially expressed and also associated with patient survival; Table S7: LincRNAs that are differentially expressed and associated with patient survival in both internal and TCGA datasets.

## References

[1] Lee P.N., Forey B.A., Coombs K.J., Lipowicz P.J., Appleton S. (2016). Time trends in never smokers in the relative frequency of the different histological types of lung cancer, in particular adenocarcinoma. Regul. Toxicol. Pharmacol..

[2] Pallis A.G., Syrigos K.N. (2013). Lung cancer in never smokers: Disease characteristics and risk factors. Crit. Rev. Oncol. Hematol..

[3] Rivera G.A., Wakelee H. (2016). Lung Cancer in Never Smokers. Adv. Exp. Med. Biol..

[4] Sun S., Schiller J.H., Gazdar A.F. (2007). Lung cancer in never smokers—A different disease. Nat. Rev. Cancer.

[5] Sun Z. (2015). High-throughput long noncoding RNA profiling for diagnostic and prognostic markers in cancer: Opportunities and challenges. Epigenomics.

[6] Derrien T., Johnson R., Bussotti G., Tanzer A., Djebali S., Tilgner H., Guernec G., Martin D., Merkel A., Knowles D.G. (2012). The GENCODE v7 catalog of human long noncoding RNAs: Analysis of their gene structure, evolution, and expression. Genome Res..

[7] Xie W., Yuan S., Sun Z., Li Y. (2016). Long noncoding and circular RNAs in lung cancer: Advances and perspectives. Epigenomics.

[8] Roth A., Diederichs S. (2016). Long Noncoding RNAs in Lung Cancer. Curr. Top. Microbiol. Immunol..

[9] White N.M., Cabanski C.R., Silva-Fisher J.M., Dang H.X., Govindan R., Maher C.A. (2014). Transcriptome sequencing reveals altered long intergenic non-coding RNAs in lung cancer. Genome Biol..

[10] Sun Z., Wang L., Eckloff B.W., Deng B., Wang Y., Wampfler J.A., Jang J., Wieben E.D., Jen J., You M. (2014). Conserved recurrent gene mutations correlate with pathway deregulation and clinical outcomes of lung adenocarcinoma in never-smokers. BMC Med. Genom..

[11] Sun Z., Nair A., Chen X., Prodduturi N., Wang J., Kocher J.-P. (2017). UClncR: Ultrafast and comprehensive long non-coding RNA detection from RNA-seq. Sci. Rep..

[12] Trapnell C., Pachter L., Salzberg S.L. (2009). TopHat: Discovering splice junctions with RNA-Seq. Bioinformatics.

[13] Pertea M., Pertea G.M., Antonescu C.M., Chang T.C., Mendell J.T., Salzberg S.L. (2015). StringTie enables improved reconstruction of a transcriptome from RNA-seq reads. Nat. Biotechnol..

[14] Trapnell C., Williams B.A., Pertea G., Mortazavi A., Kwan G., van Baren M.J., Salzberg S.L., Wold B.J., Pachter L. (2010). Transcript assembly and quantification by RNA-Seq reveals unannotated transcripts and isoform switching during cell differentiation. Nat. Biotechnol..

[15] Liao Y., Smyth G.K., Shi W. (2014). featureCounts: An efficient general purpose program for assigning sequence reads to genomic features. Bioinformatics.

[16] Love M.I., Huber W., Anders S. (2014). Moderated estimation of fold change and dispersion for RNA-seq data with DESeq2. Genome Biol..

[17] Mortazavi A., Williams B.A., McCue K., Schaeffer L., Wold B. (2008). Mapping and quantifying mammalian transcriptomes by RNA-Seq. Nat. Methods.

[18] Kim D., Langmead B., Salzberg S.L. (2015). HISAT: A fast spliced aligner with low memory requirements. Nat. Methods.

[19] Liu S., Mitra R., Zhao M.M., Fan W., Eischen C.M., Yin F., Zhao Z. (2016). The potential roles of long noncoding RNAs (lncRNA) in glioblastoma development. Mol. Cancer Ther..

[20] Li D., Li H., Yang Y., Kang L. (2017). Long noncoding RNA urothelial carcinoma associated 1 promotes the proliferation and metastasis of human lung tumor cells by regulating microRNA-144. Oncol. Res..

[21] Bian Z., Jin L., Zhang J., Yin Y., Quan C., Hu Y., Feng Y., Liu H., Fei B., Mao Y. (2016). LncRNA-UCA1 enhances cell proliferation and 5-fluorouracil resistance in colorectal cancer by inhibiting miR-204-5p. Sci. Rep..

[22] Huang J., Zhou N., Watabe K., Lu Z., Wu F., Xu M., Mo Y.Y. (2014). Long non-coding RNA UCA1 promotes breast tumor growth by suppression of p27 (Kip1). Cell Death Dis..

[23] Wang Y., Chen W., Yang C., Wu W., Wu S., Qin X., Li X. (2012). Long non-coding RNA UCA1a(CUDR) promotes proliferation and tumorigenesis of bladder cancer. Int. J. Oncol..

[24] Ulitsky I., Bartel D.P. (2013). lincRNAs: Genomics, evolution, and mechanisms. Cell.

[25] Loewen G., Jayawickramarajah J., Zhuo Y., Shan B. (2014). Functions of lncRNA HOTAIR in lung cancer. J. Hematol. Oncol..

[26] Tian X., Xu G. (2015). Clinical value of lncRNA MALAT1 as a prognostic marker in human cancer: Systematic review and meta-analysis. BMJ Open.

[27] Beane J., Vick J., Schembri F., Anderlind C., Gower A., Campbell J., Luo L., Zhang X.H., Xiao J., Alekseyev Y.O. (2011). Characterizing the impact of smoking and lung cancer on the airway transcriptome using RNA-Seq. Cancer Prev. Res..

[28] Nogueira Jorge N.A., Wajnberg G., Ferreira C.G., de Sa Carvalho B., Passetti F. (2017). snoRNA and piRNA expression levels modified by tobacco use in women with lung adenocarcinoma. PLoS ONE.

[29] Li J., Bi L., Shi Z., Sun Y., Lin Y., Shao H., Zhu Z. (2016). RNA-Seq analysis of non-small cell lung cancer in female never-smokers reveals candidate cancer-associated long non-coding RNAs. Pathol. Res. Pract..

[30] Sultan M., Amstislavskiy V., Risch T., Schuette M., Dokel S., Ralser M., Balzereit D., Lehrach H., Yaspo M.L. (2014). Influence of RNA extraction methods and library selection schemes on RNA-seq data. BMC Genom..

[31] Zhao W., He X., Hoadley K.A., Parker J.S., Hayes D.N., Perou C.M. (2014). Comparison of RNA-Seq by poly (A) capture, ribosomal RNA depletion, and DNA microarray for expression profiling. BMC Genom..

[32] Iyer M.K., Niknafs Y.S., Malik R., Singhal U., Sahu A., Hosono Y., Barrette T.R., Prensner J.R., Evans J.R., Zhao S. (2015). The landscape of long noncoding RNAs in the human transcriptome. Nat. Genet..

