# Supplementary material for: Comprehensive Profiling of lincRNAs in Lung Adenocarcinoma of Never Smokers Reveals Their Roles in Cancer Development and Prognosis
Source: Genes (Basel). 2017 Nov 13;8(11):321. doi: 10.3390/genes8110321 (PMC5704234; doi:10.3390/genes8110321)
Supplement: Supplementary file 1 [file genes-08-00321-s001.zip › Table S2.docx]

Table S2: Enriched pathways for protein coding genes that were differentially expressed where their neighboring **known** lincRNAs were also differentially expressed

| Ingenuity Canonical Pathways | -log(p-value) | Ratio |
| --- | --- | --- |
| Signaling by Rho Family GTPases | 2.93E+00 | 3.66E-02 |
| HGF Signaling | 2.14E+00 | 4.35E-02 |
| Notch Signaling | 2.14E+00 | 7.89E-02 |
| Protein Kinase A Signaling | 2.11E+00 | 2.59E-02 |
| Integrin Signaling | 2.10E+00 | 3.23E-02 |
| Mechanisms of Viral Exit from Host Cells | 2.05E+00 | 7.32E-02 |
| RhoGDI Signaling | 2.02E+00 | 3.49E-02 |
| L-cysteine Degradation II | 1.98E+00 | 1.00E+00 |
| Th1 and Th2 Activation Pathway | 1.89E+00 | 3.28E-02 |
| Phospholipase C Signaling | 1.89E+00 | 2.94E-02 |
| Neuregulin Signaling | 1.86E+00 | 4.55E-02 |
| RAR Activation | 1.82E+00 | 3.16E-02 |
| Thrombin Signaling | 1.70E+00 | 2.97E-02 |
| Th2 Pathway | 1.70E+00 | 3.38E-02 |
| Cysteine Biosynthesis/Homocysteine Degradation | 1.68E+00 | 5.00E-01 |
| Actin Nucleation by ARP-WASP Complex | 1.68E+00 | 5.36E-02 |
| Cholecystokinin/Gastrin-mediated Signaling | 1.66E+00 | 3.96E-02 |
| IGF-1 Signaling | 1.60E+00 | 3.77E-02 |
| Pyridoxal 5'-phosphate Salvage Pathway | 1.53E+00 | 4.69E-02 |
| Paxillin Signaling | 1.52E+00 | 3.57E-02 |
| Thyroid Hormone Biosynthesis | 1.51E+00 | 3.33E-01 |
