# Supplementary material for: Comprehensive Profiling of lincRNAs in Lung Adenocarcinoma of Never Smokers Reveals Their Roles in Cancer Development and Prognosis
Source: Genes (Basel). 2017 Nov 13;8(11):321. doi: 10.3390/genes8110321 (PMC5704234; doi:10.3390/genes8110321)
Supplement: Supplementary file 1 [file genes-08-00321-s001.zip › Table S5.docx]

Table S5: Enriched pathways for protein coding genes that were differentially expressed where their neighboring novel lincRNAs were also differentially expressed

| Ingenuity Canonical Pathways | -log(p-value) | Ratio |
| --- | --- | --- |
| G-Protein Coupled Receptor Signaling | 2.56E+00 | 3.66E-02 |
| Wnt/Ca+ pathway | 2.20E+00 | 6.90E-02 |
| Gαs Signaling | 1.89E+00 | 4.59E-02 |
| Dermatan Sulfate Biosynthesis (Late Stages) | 1.68E+00 | 6.52E-02 |
| RhoGDI Signaling | 1.63E+00 | 3.47E-02 |
| Chondroitin Sulfate Biosynthesis (Late Stages) | 1.61E+00 | 6.12E-02 |
| cAMP-mediated signaling | 1.61E+00 | 3.14E-02 |
| Proline Degradation | 1.60E+00 | 5.00E-01 |
| CD27 Signaling in Lymphocytes | 1.52E+00 | 5.66E-02 |
| Protein Kinase A Signaling | 1.51E+00 | 2.53E-02 |
| Chondroitin Sulfate Biosynthesis | 1.44E+00 | 5.26E-02 |
| Dermatan Sulfate Biosynthesis | 1.40E+00 | 5.08E-02 |
| ILK Signaling | 1.40E+00 | 3.06E-02 |
| IL-17A Signaling in Gastric Cells | 1.40E+00 | 8.00E-02 |
| Colorectal Cancer Metastasis Signaling | 1.40E+00 | 2.82E-02 |
| Signaling by Rho Family GTPases | 1.40E+00 | 2.82E-02 |
| IL-8 Signaling | 1.39E+00 | 3.05E-02 |
| Antioxidant Action of Vitamin C | 1.31E+00 | 3.70E-02 |
