# Supplementary figures and images for: Comprehensive Profiling of lincRNAs in Lung Adenocarcinoma of Never Smokers Reveals Their Roles in Cancer Development and Prognosis
Source: Genes (Basel). 2017 Nov 13;8(11):321. doi: 10.3390/genes8110321 (PMC5704234; doi:10.3390/genes8110321)

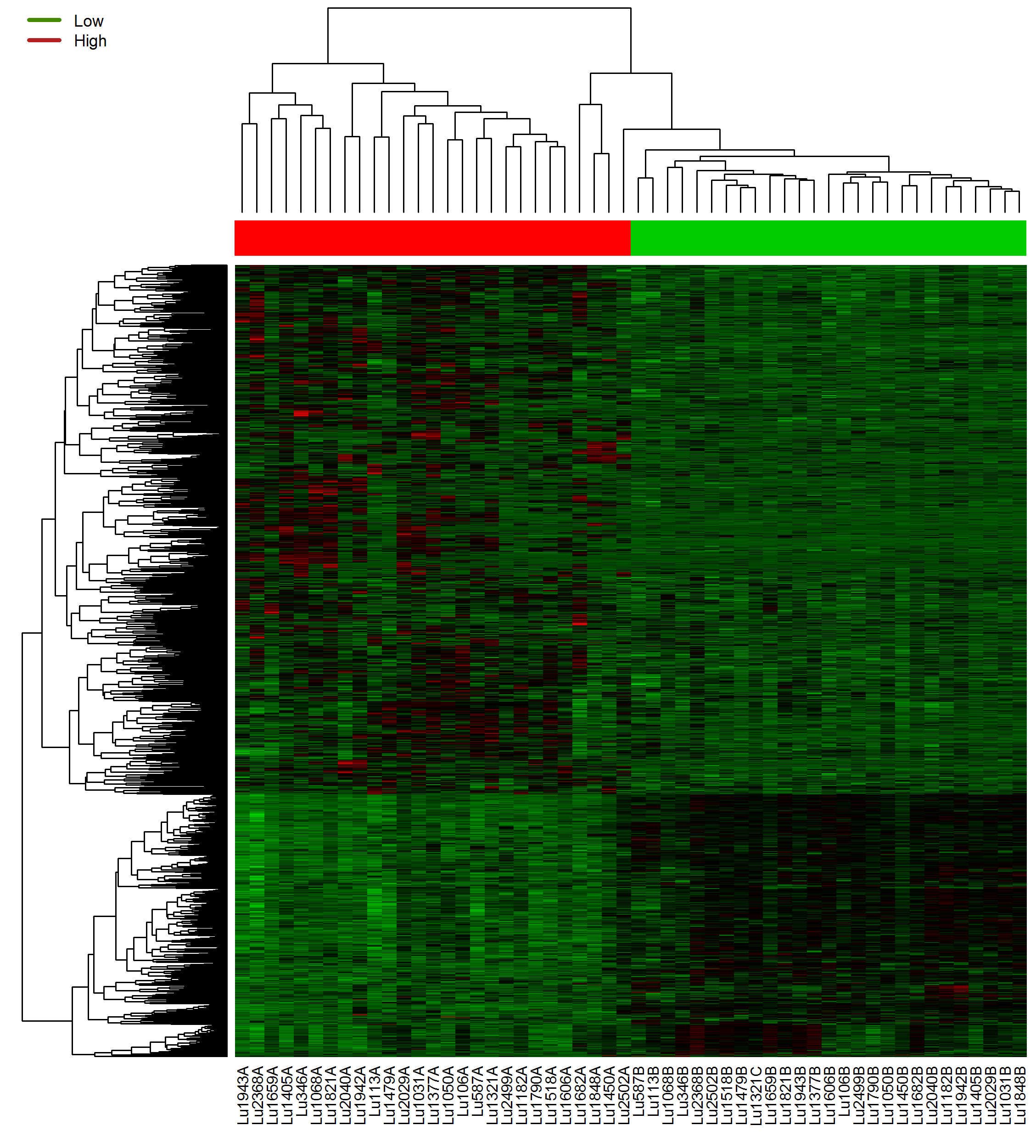

Supplement: Supplementary file 1 [file genes-08-00321-s001.zip › FigS1.png]
